# Serum glucose/potassium ratio as a clinical risk factor for predicting the severity and prognosis of acute traumatic spinal cord injury

**DOI:** 10.1186/s12891-023-07013-5

**Published:** 2023-11-09

**Authors:** Wu Zhou, Yihao Liu, Zhihua Wang, Zelu Mao, Meihua Li

**Affiliations:** Department of Neurosurgery, The First Affiliated Hospital of Nanchang University, 17 Yongwai Street, Nanchang, 330006 Jiangxi China

**Keywords:** Acute traumatic spinal cord injury, Serum glucose/potassium ratio, Biomarker, Serum glucose, Serum potassium

## Abstract

**Objective:**

Acute traumatic Spinal cord injury (TSCI) is a devastating event that causes severe sensory and motor impairments as well as autonomic dysfunction in patients, yet relevant clinical biomarkers have not been established. This study aimed to determine the significance of the serum glucose/potassium ratio (GPR) in evaluating TSCI severity and predicting prognosis.

**Methods:**

An analysis of 520 clinical records of acute TSCI patients from January 2012 to June 2022 was conducted. The relationships between serum GPR and The American Spinal Injury Association Impairment Scale (AIS) grade 6-month post-trauma prognosis and the admission AIS grade were analyzed. To evaluate the discriminatory ability, a receiver operating characteristic curve (ROC) analysis was used. All methods were performed in accordance with the relevant guidelines and regulations.

**Results:**

Based on the initial assessment of AIS grade, 256 (49.2%) patients were categorized into the severe TSCI group (AIS A–B), and there was a significant correlation between the severe TSCI group and serum GPR (*p* < 0.001). Serum GPR was reduced in an AIS grade-dependent manner (R = − 0.540, *p* < 0.001). Of the 520 patients, 262 (50.4%) patients were classified as having a poor prognosis according to the AIS grade at discharge. Serum GPR was also reduced in an AIS grade at discharge-dependent manner (R = − 0.599, *p* < 0.001), and was significantly higher in the poor prognosis group compared to the good prognosis group (*p* < 0.001). Poor prognosis was significantly associated with sex (*p* = 0.009), severity of TSCI (*p* < 0.001), location of TSCI (*p* < 0.001), surgical decompression (*p* < 0.018), body temperature (*p* < 0.001), heart rate (*p* < 0.001), systolic arterial pressure (SAP) (*p* < 0.001), diastolic arterial pressure (DAP) (*p* < 0.001), serum GPR (*p* < 0.001), serum glucose (*p* < 0.001), serum potassium (*p* < 0.001), and white blood cell count (*p* = 0.003). Multivariate logistic regression analysis showed a significant correlation between poor prognosis and serum GPR (*p* = 0.023). The ROC analysis showed the area under the curve of serum GPR to be a poor predictor of prognosis in TSCI patients at 0.842 (95% confidence interval, 0.808–0.875).

**Conclusion:**

There was a significant relationship between serum GPR and admission injury severity and the 6-month prognosis of acute TSCI patients. Serum GPR serves as a readily available clinical risk factor for predicting the severity and 6-month prognosis of acute traumatic spinal cord injury, which holds potential clinical significance for patients with TSCI.

## Introduction

In recent years, acute traumatic spinal cord injury (TSCI) has become a major global health concern, and TSCI is now considered the second leading cause after traumatic brain injury in terms of morbidity and disability [1]. Such injuries not only result in health loss and disability for individuals and their families, but also place a heavy burden on the healthcare system and the economy due to lost productivity and high medical costs [2, 3]. A global study has shown that incidence of traumatic spinal cord injuries is increasing every year, with more than 27 million people worldwide now expected to be affected by spinal cord injuries, mainly due to falls and road injuries [4]. The American Spinal Injury Association Impairment Scale (AIS) grade is used to assess the severity of TSCI and is the most common prognostic determinant of TSCI [5, 6]. However, despite an AIS classification of severe, some cases have a good prognosis. Therefore, when formulating treatment policies, it is necessary to use clinical biomarkers to predict prognosis. Our previous study showed that circulating inflammatory biomarkers (e.g., white blood cell (WBC) count and neutrophil/lymphocyte ratio) are associated with injury severity and can predict the prognosis of TSCI [7]. According to reports, inflammation biomarkers in cerebrospinal fluid (CSF) and blood (e.g., interleukin (IL)-6, IL-8, and monocyte chemotactic protein (MCP)-1), as well as structural protein biomarkers (e.g., tau, S100B, and glial fibrillary acidic protein), are useful prognostic factors; however, their detection is time-consuming and costly [8].

In general, the serum glucose level is significantly increased and serum potassium level is significantly lower after traumatic central nervous system (CNS) injury [9]. It has recently been shown that the admission serum glucose/potassium ratio (GPR) in patients with aneurysmal subarachnoid hemorrhage (SAH) is significantly associated with the Hunt and Kosnik grade and Glasgow Outcome Scale (GOS) score at discharge [10]. A retrospective study by Zhou also showed that elevated serum GPR at admission was strongly associated with 30-day mortality and Glasgow Coma Scale (GCS) in patients with traumatic brain injury (TBI) [11]. Demirtaş et al. [12] found that the GPR was significantly higher in patients who developed delayed neuropsychiatric syndrome after carbon monoxide poisoning compared to the patients without delayed neuropsychiatric syndrome, and also found that increased serum GPR may be a risk factor for the development of delayed neuropsychiatric syndrome. However, there are no studies showing the relationship between the serum GPR and the severity and prognosis of TSCI. In this study, we aimed to determine whether serum GPR could serve as a potential predictor of severity and 6-month prognosis of TSCI.

## Materials and methods

### Patients

We retrospectively analyzed clinical data related to adult TSCI patients treated at our hospital from January 2012 to June 2022. This diagnosis was confirmed by the combination of trauma history, clinical presentation, and radiographic examination. The inclusion criteria included:1) age ≥ 18 years old; 2) within 12 h following injury; 3) The Glasgow Coma Scale (GCS) = 15. The exclusion criteria were as follows: 1) pre-injury neurological deficits or related disorders (i.e. ischemic stroke, intracerebral hemorrhage); 2) Use of steroid-related drugs or diabetes; 3) kidney function injury or hematological system diseases; 4) missing data; 5)Traumatic injury to a body part other than the spinal cord with an Abbreviated score ≥ 3. In addition, control subjects required routine laboratory tests and radiologic imaging results. This study was approved by the Ethics Review Committee of the First Affiliated Hospital of Nanchang University. All methods were performed in accordance with the relevant guidelines and regulations.

### Examination, treatment and Outcome assessment

We diagnosed TSCI using magnetic resonance imaging (MRI) and physical examination on the day of admission. The clinical severity of all patients was assessed by the specialist at first contact using the AIS grade. Based on our previous study, we defined severe TSCI as AIS grades A–B and non-severe SAH as AIS grades C–E [7]. We collected clinical data, including age, sex, mechanism of injury, site of injury, admission, 6-month follow-up AIS grade, systolic blood pressure (SBP), diastolic blood pressure (DBP), heart rate, temperature, whether surgical decompression was performed, and laboratory tests. MRI was performed for all patients to determine the TSCI site. TSCI patient management and care are conducted based on the guidelines provided by the United States for treating TSCI [13]. Patient prognosis was based on the ordinal change in AIS grade after 6 months of injury. We carefully assessed the prognosis of patients with TSCI at 6 months via outpatient follow-up or by telephone consultation after discharge. The prognosis of TSCI patients who completed follow-up was classified as poor (AIS A–C) or good (AIS D–E) [6].

### Statistical analysis

SPSS (version 21.0; IBM Corp., Armonk, NY, USA) was used for all statistical analyses. Categorical variables are represented as percentages, whereas continuous variables are expressed as the mean ± standard deviation or median and interquartile range. The relationship between severe TSCI and poor prognosis with these factors was studied. The Shapiro–Wilk test was used to assess data normality. Comparisons of normally distributed variables were made using Student’s *t*-test or analysis of variance, whereas comparisons of non-normally distributed variables were made using the Kruskal–Wallis test or Mann–Whitney U-test. The Spearman’s rank correlation coefficient was used to analyze bivariate correlations. The Mann–Whitney U-test was applied to compare the GPR between poor prognosis (AIS A-C) and good prognosis (AIS D-E). Univariate analysis was used to identify statistically significant factors. Risk factors with *p* < 0.1 in the univariate model were included in the multivariate logistic regression model. The receiver operating characteristic curve (ROC) was used to find the best cut-off point for serum GPR to predict 6-month prognosis following TSCI, and the area under the curve (AUC) and 95% confidence interval (CI) were reported. Statistical differences were considered when the *p-*value < 0.05.

## Results

### Patient characteristics

There were 520 patients with acute TSCI who met the inclusion criteria (Table 1), including 430 males and 90 females, with a male-to-female ratio of 4.7:1. The age of the patients ranged from 18 to 77 (mean, 51.7) years. In general, the injury was caused mainly by falls (274, 52.7%), followed by motor vehicle accident (174, 33.5%), being struck by an object (27, 5.2%), and other injuries (45, 8.7%). The location of TSCIs in this study are shown in Table 1. Cervical TSCI accounted for 79% of all cases, and thoracic and lumbar TSCI accounted for 8.5% and 9%, respectively. According to the AIS grade, the frequencies of grade A, B, C, D, and E impairments were 198 (38.1%), 58 (11.2%), 96 (18.5%), 148 (28.5%), and 20 (3.8%), respectively. Of the total number of patients, 84.4% (439) underwent operative surgical procedures such as laminoplasty, spinal decompression, fusion, and internal fixation. After the 6-month follow-up, there were 121 (23.2%), 81 (15.7%), 60 (11.5%), 209 (40.2%), and 49 (9.4%) patients in grades A, B, C, D, and E, respectively.

**Table 1:** Patient characteristics

Variable	Value
No. of patients	520
Males/females	430(82.7%)/90(17.3%)
Mean age in years (range)	51.17(18–84)
Mechanism of injury (n, %)	
Fall	274(52.7%)
Motor vehicle accident	174(33.5%)
Struck by object	27(5.2%)
Others	45 (8.7%)
Location of injury, n (%)	
Cervical	412(79.2%)
Thoracic	44 (8.5%)
Lumbar	47 (9.0%)
Others	17 (3.3%)
AIS grade at admission	
A	198(38.1%)
B	58(11.2%)
C	96(18.5%)
D	148(28.5%)
E	20(3.8%)
Surgical decompression (N, %)	
Yes	439(84.4%)
No	81(15.6%)
6 month follow-up AIS grade	
A	121(23.3%)
B	81(15.6%)
C	60(11.5%)
D	209(40.2%)
E	49(9.4%)

### Serum GPR and severity

**Fig. 1:**
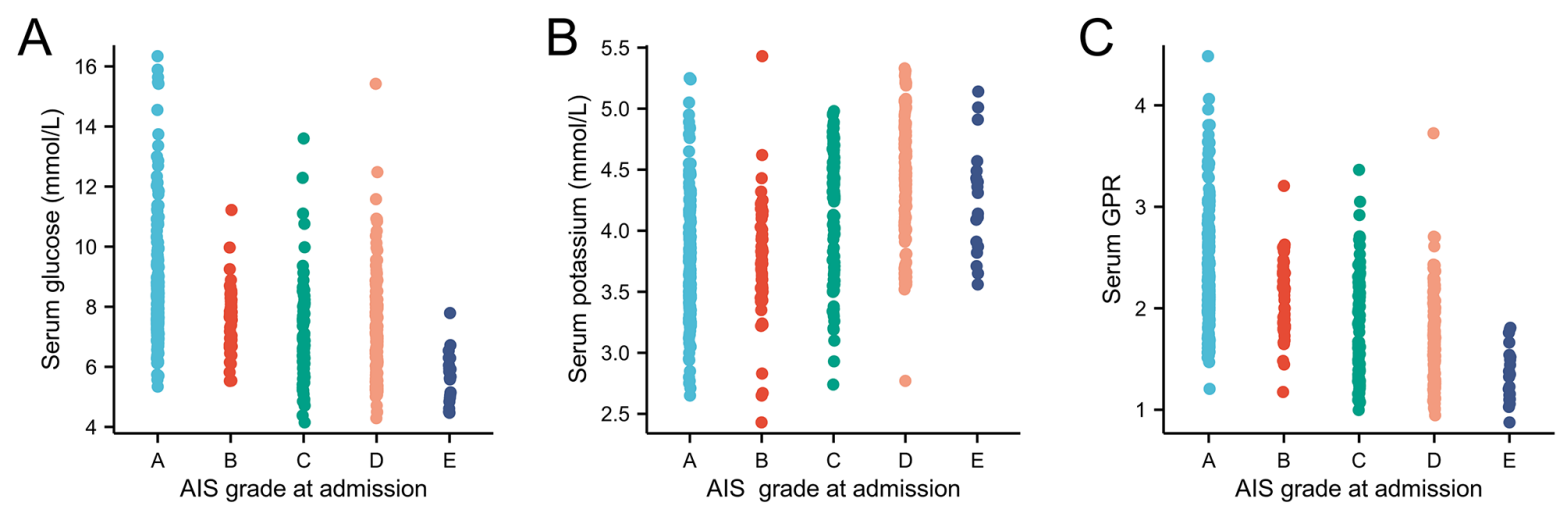
**A:** Scatterplot showing that the admission serum glucose decreased in an AIS grade-dependent manner (R=-0.410, *p* < 0.001); **B** Scatterplot showing showed that the admission serum potassium increased in an AIS grade-dependent manner (R=-0.484, *p* < 0.001); **C:** Scatterplot showing that the admission serum GPR decreased in an AIS grade-dependent manner (R=-0.540, *p* < 0.001).GPR: glucose-potassium ratio; AIS: American Spinal Injury Association Impairment Scale

Based on our previous study [7], 256 patients (49.2%) were classified as having severe TSCI (AIS A–B) and 264 patients (50.8%) were classified as having non-severe TSCI (AIS C–E) (Table 2). There were significant differences between the severe and non-severe TSCI groups in terms of sex (*p* = 0.009), location of injury (*p* < 0.001), body temperature (*p* < 0.001), heart rate (*p* < 0.001), SBP (*p* < 0.001), DBP (*p* < 0.001), serum glucose (*p* < 0.001), serum potassium (*p* < 0.001), serum GPR (*p* < 0.001), and WBC count (*p* < 0.001) (Table 2). Further investigation into the correlation between serum glucose, serum potassium, and serum GPR with the AIS grade showed that the admission serum glucose and serum GPR decreased in an AIS grade-dependent manner, while serum potassium increased in an AIS grade-dependent manner. Serum glucose (R = − 0.410, *p* < 0.001 (Fig. 1A) and serum potassium (R = − 0.484, *p* < 0.001) (Fig. 1B) were weakly correlated with AIS grade at admission, whereas serum GPR (R = − 0.540, *p* < 0.001) (Fig. 1C) was moderately correlated with AIS grade. Among them, the correlation between serum GPR and the degree of TSCI injury was highest.

**Table 2:** The differences in clinical features and laboratory data between severe and non-severe TSCI

Characteristics	Severe TSCI	Non-severe TSCI	P value
n	256	264	
Sex, n (%)			0.090
Males	219 (42.1%)	211 (40.6%)	
Females	37 (7.1%)	53 (10.2%)	
Mechanism of injury, n (%)			0.114
Fall	148 (28.5%)	126 (24.2%)	
Motor vehicle accident	77 (14.8%)	97 (18.7%)	
Struck by an object	10 (1.9%)	17 (3.3%)	
Others	21 (4%)	24 (4.6%)	
Location of injury, n (%)			< 0.001
Cervical	197 (37.9%)	215 (41.3%)	
Thoracic	33 (6.3%)	11 (2.1%)	
Lumbar	14 (2.7%)	33 (6.3%)	
Others	12 (2.3%)	5 (1%)	
age, median (IQR)	52 (43, 59)	53 (45, 60)	0.284
Body temperature (°C), median (IQR)	36.5 (36.375, 36.9)	36.7 (36.5, 37)	< 0.001
Heart rate (beats/min), median (IQR)	71 (63, 81)	77 (69, 84)	< 0.001
Breath, median (IQR)	20 (19, 20)	20 (20, 20)	0.337
Systolic arterial pressure (mmHg), median (IQR)	115 (101, 126.25)	128 (117, 139)	< 0.001
Diastolic arterial pressure (mmHg), median (IQR)	68 (59.75, 76)	78 (70, 85)	< 0.001
Serum glucose (mmol/l), median (IQR)	7.985 (7.24, 8.8025)	6.73 (5.7125, 8.0125)	< 0.001
Serum potassium (mmol/l), median (IQR)	3.68 (3.43, 4.03)	4.375 (3.925, 4.685)	< 0.001
Serum GPR, median (IQR)	2.1661 (1.8873, 2.5615)	1.5903 (1.2714, 2.0191)	< 0.001
white blood cells(10^3^/µL), median (IQR)	10.95 (9.175, 13.617)	9.69 (7.945, 12.312)	< 0.001

GPR: glucose/potassium ratio; IQR: interquartile range; TSCI: traumatic spinal cord injury; OR: Odds ratios

### Serum GPR and prognosis

**Fig. 2:**
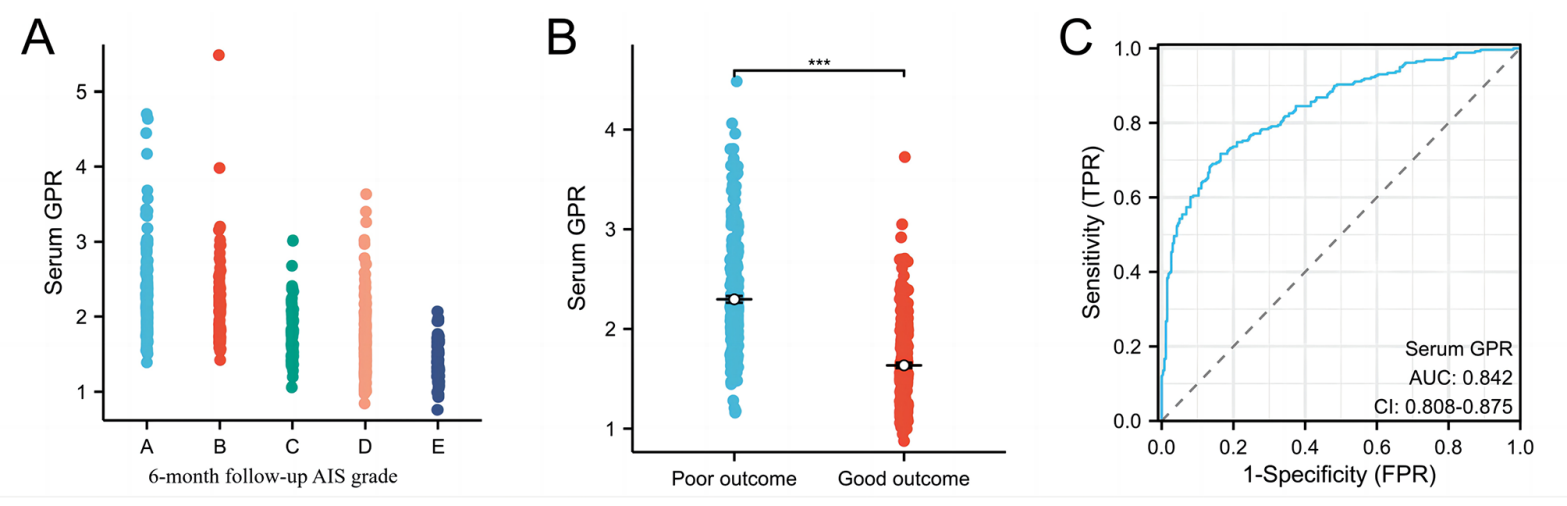
**A:** Scatterplot showing that Serum GPR increased in a AIS grade-dependent manner after a 6-month follow-up (R= -0.599, *p* < 0.001 ); **B** Scatterplot showing that serum GPR with good prognosis group was significantly lower than in the poor prognosis group (*p* < 0.001); C:The ROC curve analysis of serum GPR for predicting poor prognosis. The AUC of serum GPR was 0.842 (95%CI 0.808–0.875). AUC: area under the curve; GPR: glucose-potassium ratio; AIS: American Spinal Injury Association Impairment Scale; ROC: receiver operating characteristic

According to the assessment of the AIS grade at 6 months after injury, 262 patients (49.2%) were classified as having a poor prognosis (AIS A–C) and 258 patients (50.8%) were classified as having a good prognosis (AIS D–E) (Table 3). Serum GPR increased in a AIS grade-dependent manner after a 6-month follow-up (R = − 0.599, *p* < 0.001) (Fig. 2A). The univariate analysis indicated that sex, severity of injury, location of injury, SBP, DBP, heart rate, surgical treatment, serum glucose level, serum potassium level, serum GPR, and WBC count were significantly associated with the 6-month prognosis. We also found that the serum GPR of TSCI patients with a good prognosis was significantly lower than that of TSCI patients with a poor prognosis (Fig. 2B). To analyze and adjust for multiple predictors, we performed multivariate logistic regression analyses (Table 4). We found that serum GPR remained a significant prognostic factor affecting the 6-month prognosis of TSCI patients (odds ratio (OR), 0.001 (0.000–0.165), *p* < 0.001), whereas serum glucose and serum potassium were not significantly associated with the 6-month prognosis. In addition, the severity of TSCI at admission was an independent predictor of the 6-month prognosis (OR, 0.009 (0.004–0.021)). Finally, to assess the serum GPR prediction performance, we constructed an ROC and calculated the AUC. The ROC analysis showed the AUC of serum GPR to be a good predictor of prognosis in TSCI patients at 0.842 (95% CI, 0.808–0.875) (Fig. 2C).

**Table 3:** Results of statistical analysis of the risk factors for poor prognosis in TSCI

Characteristics	Poor outcome	Good outcome	P value
n	262	258	
Sex, n (%)			0.009
Males	228 (43.8%)	202 (38.8%)	
Females	34 (6.5%)	56 (10.8%)	
Mechanism of injury, n (%)			0.770
Fall	143 (27.5%)	131 (25.2%)	
Motor vehicle accident	85 (16.3%)	89 (17.1%)	
Struck by object	21 (4%)	24 (4.6%)	
Others	13 (2.5%)	14 (2.7%)	
Severity of TSCI, n (%)			< 0.001
Severe TSCI	239(46%)	17(3.3%)	
Non-severe TSCI	23(4.4%)	241(46.3%)	
Location of TSCI, n (%)			< 0.001
Cervical	204 (39.2%)	208 (40%)	
Thoracic	11 (2.1%)	36 (6.9%)	
Lumbar	12 (2.3%)	5 (1%)	
Others	35 (6.7%)	9 (1.7%)	
Surgical decompression, n (%)			0.018
Yes	231 (44.4%)	208 (40%)	
No	31 (6%)	50 (9.6%)	
age, median (IQR)	52 (44.25, 59.75)	53 (45, 59.75)	0.839
Body temperature (°C), median (IQR)	36.5 (36.4, 36.9)	36.7 (36.5, 37)	0.007
Heart rate (beats/min), median (IQR)	72 (64, 80)	77 (69, 84)	< 0.001
Breath, median (IQR)	20 (19, 20)	20 (20, 20)	0.236
Systolic arterial pressure (mmHg), median (IQR)	116 (100, 128)	127 (116, 137.75)	< 0.001
Diastolic arterial pressure (mmHg), median (IQR)	68 (59, 77)	77 (70, 85)	< 0.001
Serum glucose (mmol/l), median (IQR)	7.595 (6.77, 8.6775)	6.225 (5.31, 7.32)	< 0.001
Serum potassium (mmol/l), median (IQR)	3.71 (3.49, 4)	4 (3.75, 4.22)	< 0.001
Serum GPR, median (IQR)	2.0533 (1.7893, 2.3893)	1.5381 (1.3462, 1.7877)	< 0.001
white blood cells(10^3^/µL), median (IQR)	10.825 (9.075, 13.652)	9.865 (7.935, 12.403)	0.003

CI: confidence interval; GPR: glucose/potassium ratio; IQR: interquartile range; TSCI: traumatic spinal cord injury; OR: Odds ratios

**Table 4:** Multivariate logistic regression analysis for risk factors of poor outcome in TSCI

Characteristics	OR (95% CI)	P value
Sex, n (%)	0.432(0.161–1.443)	0.192
Severity of TSCI	0.009 (0.004–0.021)	< 0.001
Location of injury		0.227
Surgical decompression	1.387 (0.491–3.918)	0.537
Heart rate	1.001 (0.971–1.031)	0.967
Systolic arterial pressure	1.008 (0.977–1.041)	0.611
Diastolic arterial pressure	0.973 (0.925–1.023)	0.279
Serum glucose	0.236 (0.043–1.289)	0.096
Serum potassium	0.163 (0.005–4.911)	0.371
Serum GPR	0.000 (0.000–0.324)	0.023
White blood cells	1.026 (0.922–1.141)	0.638

CI: confidence interval; GPR: glucose/potassium ratio; TSCI: traumatic spinal cord injury; OR: Odds ratios

## Discussion

This is the first study to examine the correlation of serum GPR with injury severity and 6-month prognosis for patients with TSCI. In this study, serum GPR in patients with TSCI was significantly correlated with the severity of injury at admission. We also demonstrated that serum GPR was strongly associated with a poor prognosis. In addition, serum GPR was significantly elevated in the severe TSCI group with a poor prognosis compared with the good prognosis group. Thus, serum GPR shows potential as a biomarker of the severity of TSCI and for predicting the prognosis of TSCI patients.

Efforts are ongoing to find simple, accessible prognostic predictors of TSCI to optimize therapeutic decisions. The AIS grade has been widely used as the most acceptable predictor of prognosis after TSCI [14, 15]. Our study confirmed that admission TSCI injury severity (AIS grade) was an independent significant predictor of prognosis for TSCI patients. However, there are some shortcomings when applying AIS grade in clinical work. First, there is considerable uncertainty for patients with spinal shock in general, as it is widely accepted that some degree of spontaneous recovery could occur within several hours of injury. Second, more consideration should be given to the heterogeneity of the patient population with TSCI, which can have an important influence on the results and the under-representation of such factors in current clinical studies. Therefore, further research is needed to develop diagnostic and predictive prognostic models for acute TSCI, and a framework for identifying valuable biomarkers is also necessary to improve the accuracy of prognostic prediction. Some inflammatory biomarkers, such as C-reactive protein, IL-6, or MCP-1, participate in the systemic inflammatory response and can be transported into the blood or CSF [8, 16, 17]. Circulating blood and CSF structural protein biomarkers like total tau, S100B, glial fibrillary acidic protein, and neuron-specific enolase have attracted attention for assessing the prognosis and severity of TSCI [8, 18, 19].Additional potential peripheral blood and CSF biomarkers such as neurofilament heavy chain, neurofilament light chain, CCL4, and C-X-C chemokine ligand (CXCL)-1, CXCL9, and CXCL12 can also be used to assess the severity of TSCI and predict recovery outcomes [20]. However, due to the time required and monetary costs, the measurement of these biomarkers tends to be inconvenient. Thus, due to the lack of clinical feasibility, these detection methods have not been widely applied. Therefore, the search for new biomarkers is an effective way for clinicians to have access to more patient-specific information, and these data can be easily integrated into clinical treatment protocols, especially during the management of acute TSCI patients. Thus, the development and validation of new prognostic models based on biomarkers readily available in daily clinical practice will help predict neurological outcomes in TSCI. Currently, A number of simple and low-cost routine biochemical markers at admission include serum glucose and potassium are well known.

Catecholamines are critical after injury and stress, because they not only promote glucagon secretion but also inhibit insulin secretion, which further increases serum glucose level [21]. Ogura showed that sympathetic activation in patients with acute neurological diseases produces excessive catecholamines and is strongly correlated with disease severity [22]. Moreover, animal studies have shown that endoplasmic reticulum stress in rats is enhanced with increasing serum glucose level, leading to more severe blood-spinal cord barrier disruption. This results in increased neuronal cell apoptosis, glial cell proliferation, and secretion of inflammatory factors, further interfering with movement recovery [23]. Other reports have indicated that the enhancement of endoplasmic reticulum stress under elevated serum glucose level serves as a significant risk factor for secondary damage following primary spinal cord injury, this state may impede neurogenesis and result in detrimental effects on CNS repair [24, 25]. The serum glucose level correlates with patient clinical condition and has been reported to predict poor prognosis after TSCI. Additionally, Studies have also shown elevated serum glucose to be a detrimental factor in the restoration of motor function in patients with cervical spinal cord injury [26].Therefore, it is reasonable that an elevated serum glucose level is highly correlated with the severity of CNS injury and post-injury prognosis, as confirmed in several previous reports. However, the association between serum glucose concentration and the 6-month prognosis in TSCI was found only in univariate analyses and not in multivariate models in our study.

Most of the potassium in the human body (98%) is stored intracellularly, with potassium being actively taken up and transported across the cell membrane via the adenosine triphosphatase sodium/potassium pump (Na^+^/K^+^-ATpase). Na^+^/K^+^-ATpase is regulated by catecholamines, B2 adrenergic hormones, and insulin, which lead to reduced serum potassium level [27]. Epinephrine also reduces serum potassium level, because it is stimulated by β-adrenergic receptors linked to membrane Na^+^/K^+^-ATpase, resulting in potassium influx into the intracellular space [28]. Acute TSCI produces excessive catecholamine secretion and elevates serum glucose level [29, 30]; therefore, this condition promotes insulin secretion and the entry of serum potassium into cells, further decreasing serum potassium level. Animal studies have indicated that potassium channels may mediate changes in the properties of reticular neurons after spinal cord injury. Another animal study found that after spinal cord injury, neuronal death and demyelination resulted in the activation of potassium channels, leading to a decrease in the compound action potential amplitude and axonal response to high-frequency stimulation within the injured spinal cord, ultimately reducing axonal conduction [31]. Based these findings, we hypothesize that TSCI exacerbates secondary damage caused by decreased serum potassium.

Reports have indicated that elevated glucose and electrolyte imbalance after spinal cord injury are associated with early mortality and poor prognosis [32, 33]. Combining our study results, we believe that an elevated GPR in TSCI patients can partially reveal a poor prognosis. According to Fujiki et al. [10], serum glucose, serum potassium, and serum GPR in patients with aneurysmal SAH were significantly correlated with the Hunt–Hess grade, as well as the 3-month post-discharge GOS score. Zhou et al. [11] showed that serum GPR in severe TBI patients was strongly correlated with trauma severity and 30-day mortality. Jung et al. [34] also demonstrated that higher plasma GPR at admission is a potential predictor of 3-month mortality in patients with aneurysmal SAH. Similarly, our study showed that a high serum GPR at admission was associated with adverse outcomes in patients with TSCI. This suggests that in the early stage of TSCI, close attention should be paid to the serum glucose and electrolyte (especially potassium ion) level of patients. Maintaining normal serum glucose level and electrolyte balance during the early stages of trauma may play a vital role in patient prognosis.

### Limitation

This study has several limitations. 1). We did not observe the endocrine level like.

catecholamines, glucagon, corticosteroids, and insulin; thus, the true cause of high serum GPR in patients with severe TSCI is unknown. 2).This study is a single-center retrospective design with a relatively small sample size, which may lead to selection bias and inability to control all confounding factors. 3). Serum glucose and potassium level may be affected by a number of factors, including the timing of pre-injury food intake and the use of some medications that affect serum glucose and potassium level, but we did not account for these factors. 4). Other important inflammatory biomarkers such as CRP may also be associated with TSCI prognosis, however this study did not make a comparison.

## Conclusion

To our knowledge, our study is the first to demonstrate the close association of the serum GPR with the severity and 6-month prognosis of acute TSCI. Serum GPR serves as a readily available clinical risk factor for predicting the severity and 6-month prognosis of acute TSCI, which holds potential clinical significance for patients with acute TSCI. Further validation of our findings is required through larger-scale studies.

## Data Availability

The datasets used and/or analysed during the current study available from the corresponding author on reasonable request.

## Abbreviations

AIS: American spinal injury association Impairment Scale
AUC: Area under the curve
BSCB: Blood-spinal cord barrier
CI: Confidence interval
CNS: Central nervous system
CRP: C-reactive protein
DBP: Diastolic blood pressure
GCS: Glasgow Coma Scale
GOS: Glasgow Outcome Scale
GPR: Glucose/potassium ratio
ROC: Receiver operating characteristic
SAH: Subarachnoid hemorrhage
SBP: Systolic blood pressure
SD: Standard deviation
TBI: Traumatic brain injury
TSCI: Traumatic spinal cord injury
WBC: White blood cell
